# Mobile Technology for Empowering Health Workers in Underserved Communities: New Approaches to Facilitate the Elimination of Neglected Tropical Diseases

**DOI:** 10.2196/publichealth.5064

**Published:** 2016-01-14

**Authors:** Michelle Stanton, Andrew Molineux, Charles Mackenzie, Louise Kelly-Hope

**Affiliations:** ^1^ Liverpool School of Tropical Medicine Department of Parasitology Liverpool United Kingdom; ^2^ Tripod Software Limited Salford Innovation Forum Manchester United Kingdom

**Keywords:** mhealth, lymphatic filariasis, LF, elephantiasis, neglected tropical diseases, NTDs, community engagement, SMS, smartphones, apps

## Abstract

**Background:**

As global mobile phone penetration increases, direct health information communication from hard-to-reach communities is becoming commonplace. Mobile health (mHealth) tools that enable disease control programs to benefit from this information, while simultaneously empowering community members to take control of their own health, are vital to the goal of universal health care.

**Objective:**

Our aim was to highlight the development of the Liverpool mHealth Suite (LMS), which has been designed to address this need and improve health services for neglected tropical diseases being targeted for global elimination, such as lymphatic filariasis.

**Methods:**

The LMS has two main communication approaches—short message service and mobile phone apps—to facilitate real-time mass drug administration (MDA) coverage, reporting patient numbers, managing stock levels of treatment supplies, and exchanging health information to improve the quality of care of those affected.

**Results:**

The LMS includes the MeasureSMS-MDA tool to improve drug supplies and MDA coverage rates in real-time (currently being trialed in urban Tanzania); the MeasureSMS-Morbidity tool to map morbidity, including lymphedema and hydrocele cases (initially piloted in rural Malawi and Ghana, then extended to Ethiopia, and scaled up to large urban areas in Bangladesh and Tanzania); the LyMSS-lymphedema management supply system app to improve distribution of treatments (trialed for 6 months in Malawi with positive impacts on health workers and patients); and the HealthFront app to improve education and training (in development with field trials planned).

**Conclusions:**

The current success and scale-up of the LMS by many community health workers in rural and urban settings across Africa and Asia highlights the value of this simple and practical suite of tools that empowers local health care workers to contribute to local, national, and global elimination of disease.

## Introduction

A well-tested approach to achieving major health improvements for less fortunate and underserved people in developing countries is to actively involve the local community in the management, distribution, and advocacy of much-needed health practices [1,2]. A major example of this is seen in the world of parasite infection control with the very successful involvement of village personnel in the mass drug administration (MDA) activities of the anti-filarial chemotherapeutic agent, ivermectin, used for onchocerciasis (river blindness). This was termed “community-directed treatment with ivermectin” [3]. The direct involvement of the affected communities and those working in health within these communities (eg, village health workers, community drug distributors [CDDs]) has been an essential part of the global success of this and similar filarial disease programs such as lymphatic filariasis (LF) [4,5].

Here we present an additional way of empowering the local communities using current mobile phone technology developed at the Liverpool School of Tropical Medicine (LSTM) to specifically improve and enhance the implementation of national filarial disease programs. Mobile phones are arguably the most sophisticated form of modern technology to become universally available to underprivileged populations of the world, and the trend of using mobile phones to improve health services (mHealth) is rapidly increasing [6-9]. Mobile phone technology has to date been used in a wide range of health settings including data collecting and reporting, decision-making support for health workers, and delivering health information to the public (eg, appointment reminders, health quizzes).

## Methods

### The Liverpool mHealth Suite

The success of the tools we have developed—the Liverpool mHealth suite (LMS)—is attributed to the use of both local health workers’ knowledge and their own mobile phone handsets to generate primary data on treatment and morbidity. This has helped enhance the quality of health care they provide. The LMS tools focus on the parasitic disease LF (elephantiasis), which is one of the 17 neglected tropical diseases reported by the World Health Organization (WHO) and targeted for global elimination [4]. The LMS has been developed directly to enhance the mass drug administration (MDA) and patient care, currently referred to as morbidity management and prevention, components of the Global Programme to Eliminate Lymphatic Filariasis, as well as help prepare endemic countries for successful elimination verification.

### Main Communication Approaches

**Figure 1:**
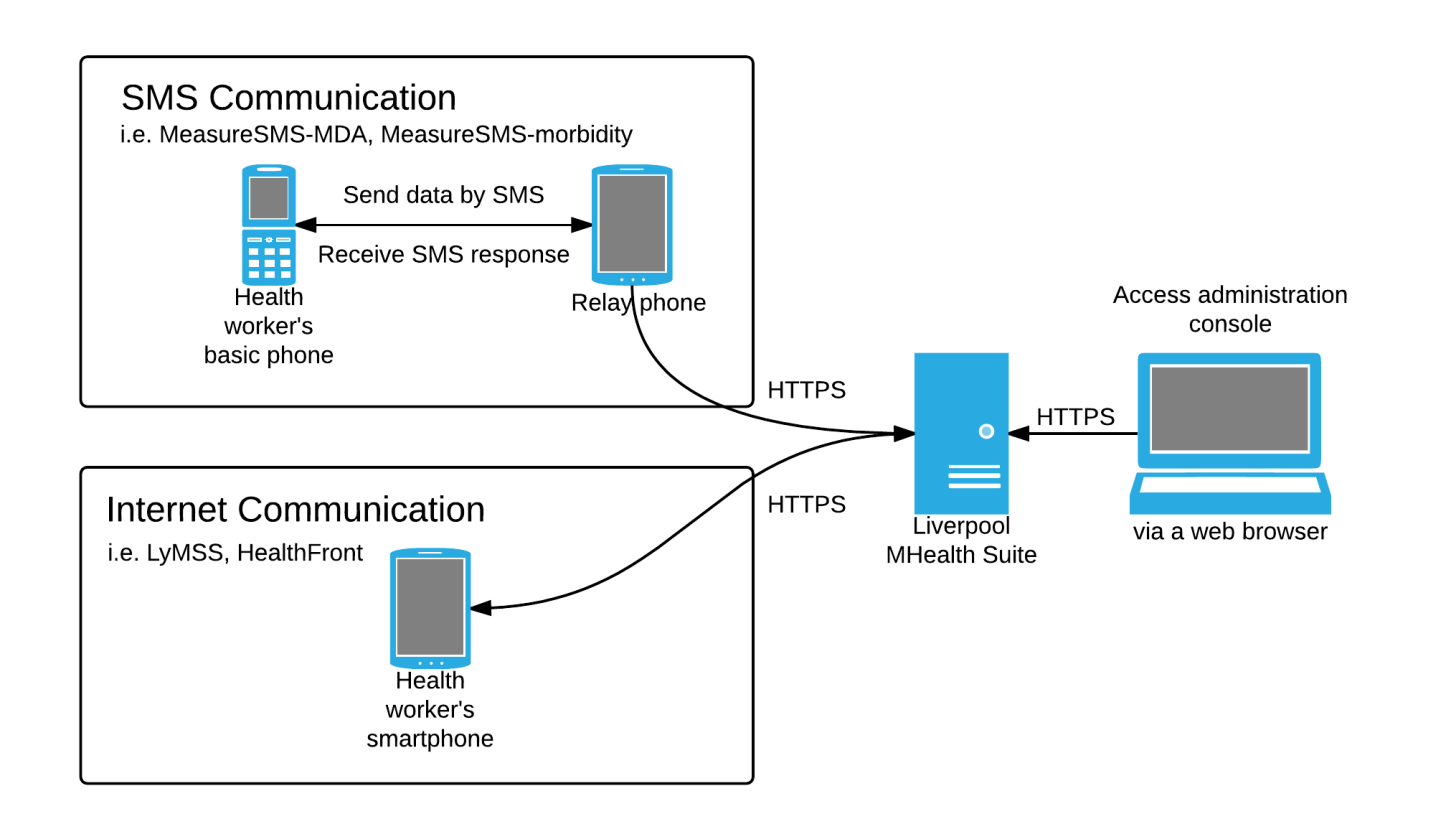
The two main approaches of the Liverpool mHealth Suite (LMS).

We have adopted two main communication approaches to undertake these tasks (see Figure 1): short message service (SMS) communication and Internet communication.

SMS tools allow village health workers using readily available mobile phones with basic functionality (ie, not necessarily smartphones), which they use to send information, in the format of a standard SMS, to a local phone number. This SMS is received by a locally situated smartphone, which plays the role of the local server. We refer to this as the relay phone. The relay phone transmits the information to a central cloud-based server via the Internet using either WiFi or a local mobile Internet connection (via https) and further sends feedback to the health workers by SMS. Data received by the central server can be accessed via a Web browser, allowing real-time field collected data status, treatment needs, and other information of health activities occurring at the community level to be accessed in real-time.

Internet tools allow health workers to send and receive information (eg, text, pictures, location information) using mobile phone–based apps that are accessed via a local Internet connection. The health workers’ mobile phones communicate directly with the central server to facilitate the provision of essential medical care and supplies to remote locations in a timely manner. This also helps to instantly obtain current information on best care medical practices.

Both the SMS component and Internet component of the LMS differ from many of the mobile technology approaches previously developed. Formerly, these have been mainly top-down approaches where the communication is initiated by external or senior-level investigators, rather than being used by local health workers in the field. The LMS tools have been applied in both rural and urban settings by local health workers to acquire essential data that address four important national programmatic areas relating to the elimination of LF disease (see Table 1).

The first two programmatic areas use the MeasureSMS system to collate and report MDA treatment numbers (MeasureSMS-MDA) and morbidity information (MeasureSMS-Morbidity), including lymphedema and hydrocele cases, at the village and health center levels [10]. The second two programmatic areas make use of mobile phone apps to coordinate the distribution of lymphedema management supplies (LyMSS-lymphedema management supply system) and provide the most up-to-date health information to frontline health workers (HealthFront) and their supervisors. Details of each of these are outlined below.

**Table 1:** Summary of the tools included in the Liverpool mHealth suite.

No.	Name of tool	Mode	Description
1	MeasureSMS-MDA	SMS	Reporting MDA treatment numbers and preliminary clinical case data at health facility level to help improve drug distribution and population coverage rates.
2	MeasureSMS-Morbidity	SMS	Reporting clinical case numbers, age, sex, condition, severity, and acute attacks at village and health facility level to help improve management and disability prevention services.
3	LyMSS	Internet	Maintaining a supply chain of morbidity care packages including wash basins, towels, soaps, and antibacterial creams to help ensure the provision of basic care to patients.
4	HealthFront	Internet	Providing up-to-date practical health information to health facility workers for managing clinical conditions to help improve the conditions and quality of life of patients.

## Results

### Areas of Application

#### Tool 1. Monitoring Mass Drug Administration Treatment Numbers and Preliminary Morbidity Case Detection

**Figure 2:**
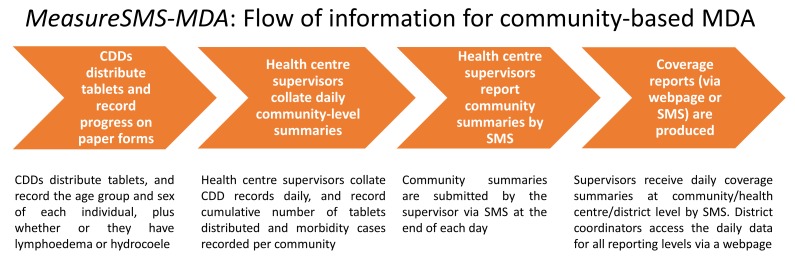
Flow of information using the MeasureSMS-MDA system.

The MeasureSMS-MDA reporting tool is being used for real-time reporting of the number of people treated during MDA campaigns, plus the number of morbidity cases seen during the campaign (see Figure 2). Health facility/distribution post level treatment numbers, including the number of tablets distributed, and patient numbers are submitted to the MeasureSMS-MDA system via SMS by health workers at the end of each day of an MDA campaign, and these numbers are instantaneously viewable in a database via a Web browser. Health workers immediately receive an SMS message, which acknowledges both the receipt of their report, plus provides feedback on the progress of the campaign in their area (eg, reported cumulative coverage in relation to target coverage, or the number of tablets remaining at the facility). This real-time information sharing empowers both the health worker and MDA supervisors at the district or national level. The health worker, in receiving instant feedback on their progress, is able to determine how to more effectively manage their campaign to meet coverage targets. Further, the knowledge that their activities are being reviewed by more senior campaign members may lead them to feel more connected to the MDA program and thus more motivated to ensure its success. The MDA supervisors are empowered through gaining instant access to data, which will help them identify facilities or distribution posts that need additional assistance proactively rather than retroactively (eg, helping facilities acquire more tablets before their stock runs out).

The use of this tool is particularly important for countries starting or scaling up MDA implementation. Post-MDA treatment summaries can further help identify areas where drugs need to be targeted to improve coverage and help interrupt transmission. Further, it can provide a crude baseline of clinical case numbers on which to build more detailed information. This is important where there is a lack of information on disease prevalence. Figure 2 describes the flow of information during an MDA campaign in a rural setting where reports are made at health facility level and CDDs distribute tablets door to door.

#### Tool 2. Morbidity Mapping and Epidemiology

**Figure 3:**
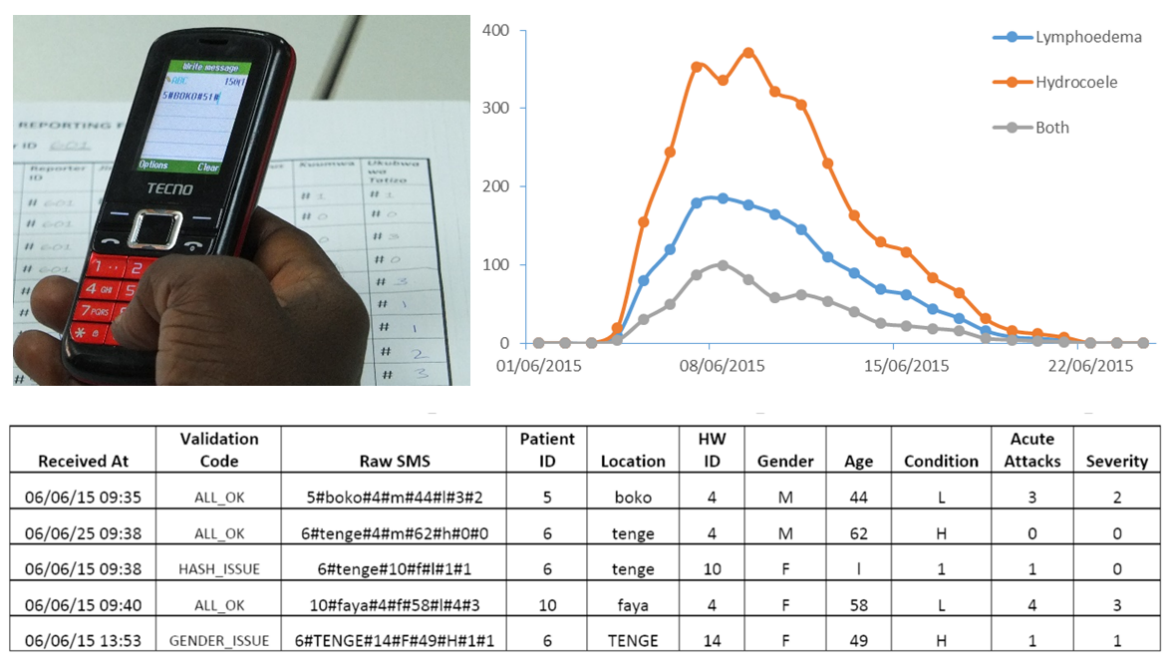
The MeasureSMS-Morbidity tool data entry and presentation.

The MeasureSMS-Morbidity reporting tool is used to improve information on LF patient numbers, including location, age, sex, clinical condition (ie, lymphedema, hydrocele), and details about severity of condition (ie, mild, moderate, severe) and episodes of acute attacks [10]. Data are sent in as a simple SMS message (one message per patient) by health workers during a community cross-sectional survey and are collated into a national disease database that is accessible by national and district health teams via a Web browser, secured with a username and password. The number of cases reported can be viewed in real-time, allowing the progress and quality of data to be monitored during the survey period. Figure 3 presents an example of a health worker writing an SMS message containing example patient data, as well as examples of the data that can be accessed via the MeasureSMS-Morbidity webpages (ie, a time series plot of reported cases) and a spreadsheet of the resulting data. To verify the quality of the reporting in relation to clinical condition and severity, a random sample of individuals (with sample size based on estimated positive predictive value, eg, [10]) are followed up by the district health team and medical doctor. This tool again empowers both the health workers who report the data and the individuals who oversee the data reporting by increasing the sense of data ownership and accountability, and opening the channels of communication between the health workers and the patients, plus the health workers and those overseeing the activity.

This tool is important in addressing the major challenge of estimating patient numbers in each endemic country and will help national LF elimination programs provide basic health care for those suffering from this disease [11]. The identification and management of cases needing surgery for hydrocele and care for lymphedema is also greatly improved by using MeasureSMS-Morbidity. Further, if implemented on a regular basis, the MeasureSMS-Morbidity system can be used to detect new cases and monitor the decline in prevalence over time. Case number data have become increasingly important and are now formally required by the WHO for the official verification. Thus, the MeasureSMS-Morbidity tool gives the community members a key role in contributing to the national programmatic success.

#### Tool 3. Delivery of Care to Patients

**Figure 4:**
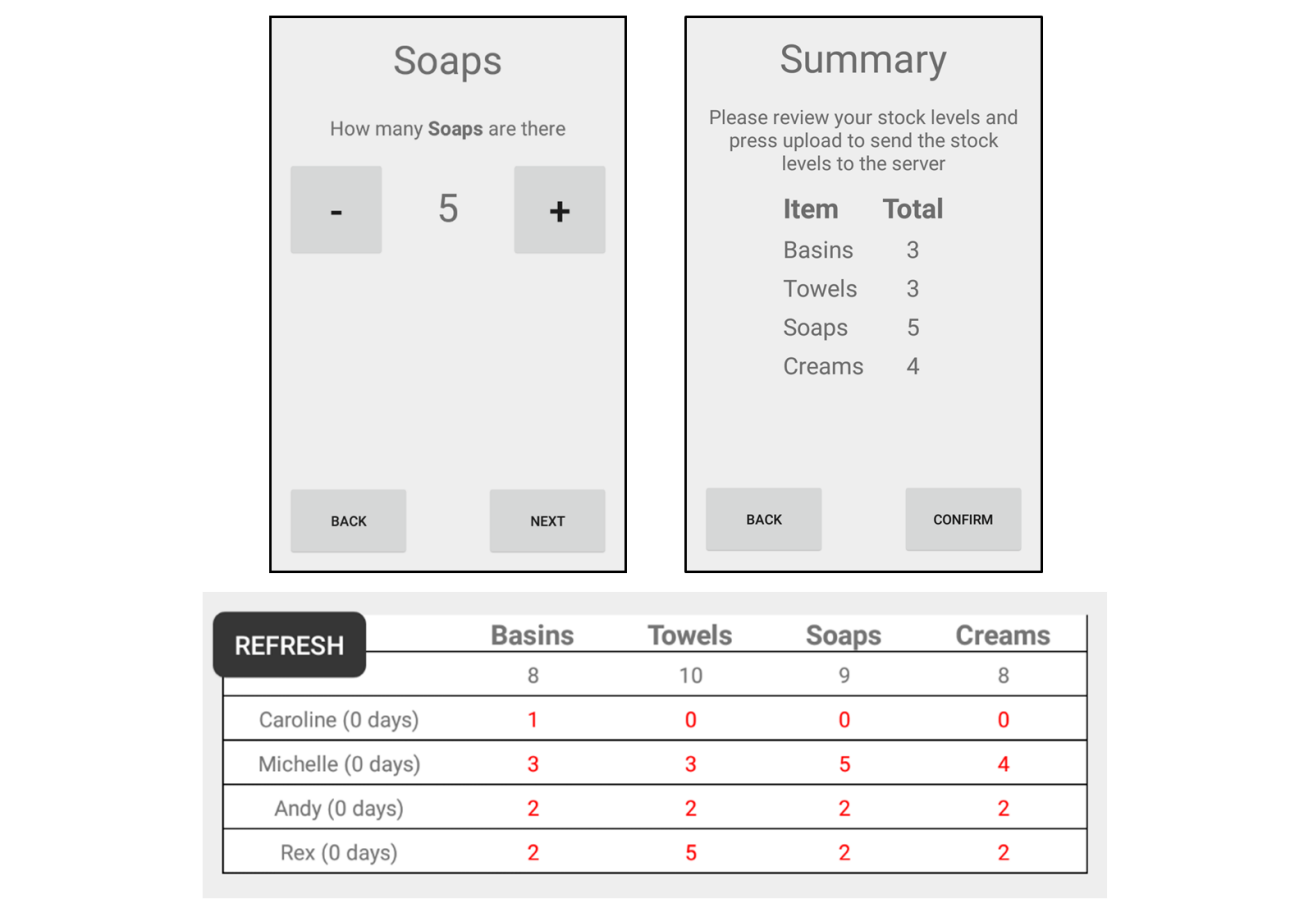
An example of the LyMSS stock entry app.

LyMSS is a lymphedema management supply system that makes use of mobile phone apps to help provide basic care packages (ie, towels, soap, antibacterial creams [12]) to those afflicted with the chronic disease and disfigurement that occur in LF patients. Currently, there is no sure way for central management and supply offices to know the local supply needs/demand or locally held basic care inventory (ie, by patients, community health workers, health centers) at a particular point in time. The LyMSS app allows health workers to submit regular inventory reports, which are instantly viewable by their local supply managers on their own mobile phones and by the national-level supply managers via a Web browser. Figure 4 presents an example of the supply manager app and reported levels of soaps in one of the study areas over a 6-month period. This information can then be used to direct supplies to where they are most needed in a timely manner and thus ensure patients have continuous access to the items that they need to effectively care for their condition. This system empowers the individuals involved in reporting and overseeing the stock levels to take ownership of the supply chain. The system does not dictate when supplies need to be distributed, as during its development it was acknowledged that the LyMSS users will have the most accurate knowledge of their own distribution rates and should therefore be encouraged to determine how and when the supply manager should supplement their supplies. The method and frequency of delivering supplies to patients was also determined by the local health workers to ensure these activities could fit easily into their usual activities.

While SMS-based stock management approaches have been shown to be successful in a number of applications [13,14], these tools are limited in their functionality. As smartphones are likely to become increasingly ubiquitous in even the most remote of settings [15], it is important to develop applications that use smartphones and thus extend the approach to activities that are not possible under an SMS model (eg, recording photographs of the patient’s clinical condition). However, as not all locations are expected to have mobile data coverage at present, or data coverage may too expensive, a more basic SMS reporting approach may be more suitable for supply management purposes in these locations in the interim.

The LyMSS tool will become increasingly more important as morbidity management activities become the main focus of the LF elimination programs in endemic countries. The increased need for the provision of the basic kits and supplies from international sources will be especially important for community/home-based lymphedema management. It will be critical to keep track of the community needs to ensure the supply meets local demands. In a wider context, the availability and access to health care, aside from being one of the WHO criteria for countries to achieve elimination [12], is also a goal of the wider principle of strengthening health care access for all [16].

#### Tool 4. Health Information for Best Care Practice

HealthFront is another vital LMS tool currently under development, which involves the provision of practical health information to health workers for decision making, particularly for those who are at the patient-health interface for LF and other neglected tropical diseases. Ready access of current practical information to health workers on clinical management will improve patients’ health, as well as empower their status and ability. This new LMS tool will deliver regularly updated information to health workers. It will work most efficiently and effectively as a smartphone app in order to support more visual information in a user-friendly format. A simpler text-only based approach is also being considered to complement the smartphone app in order to accommodate those without smartphone access, thus ensuring that access to the most sophisticated mobile technology is not a barrier to health information and quality of care.

### Recent Epidemiological and Program Implementation

**Figure 5:**
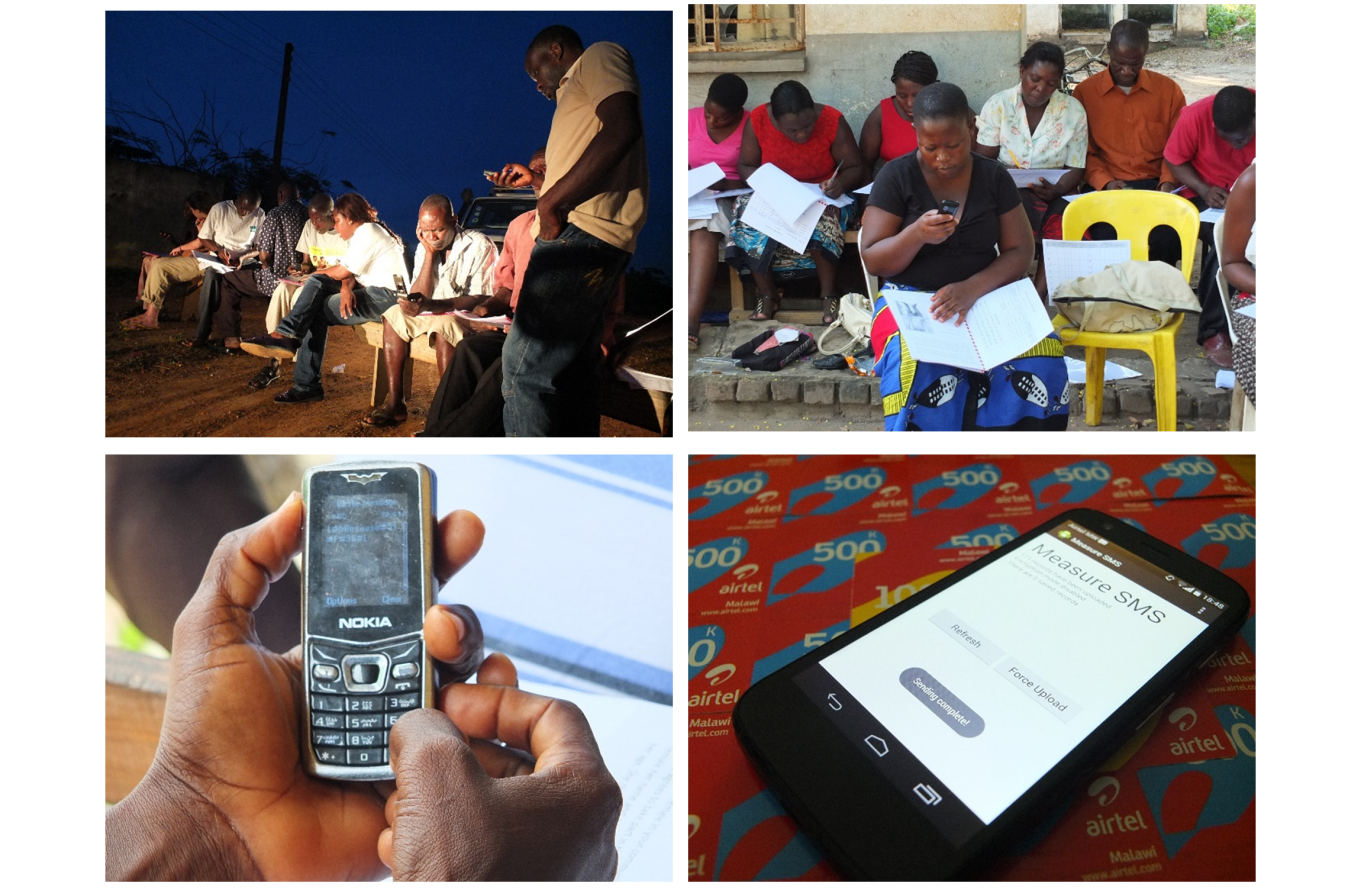
An example of the MeasureSMS-Morbidity training workshop.

MeasureSMS-Morbidity has been extensively field tested in both rural and urban environments, providing vital LF patient baseline data (Figure 5) [10]. In brief, in 2014 local health surveillance assistants (HSAs) in parts of Chikwawa district, Malawi (covering a population of 107,000), and volunteer community health workers (CHWs) in parts of Ahanta West district, Ghana (covering a population of 45,000), used the tool to identify LF lymphedema and hydrocele patients in their own health catchment areas. Although there was little difference in mobile phone ownership, large differences in mobile phone experience were observed between these two reporting groups: 100% (60/60) in Malawi had used their phones to send SMS text messages compared to 44% (14/32) in Ghana. Due to the nature of their work, all health workers involved were very familiar with community members in their catchment area, and patient identification was undertaken using a mixture of methods including prior knowledge, community gatherings, and household visits.

Post-study questionnaires were completed by the majority of health workers involved in both surveys, in which they were asked to identify any difficulties they had in undertaking the survey and further highlight the perceived benefits. In Malawi, 96% (43/45) of HSAs reported that they found it easy to submit data by SMS, stating that the greatest benefits were that information could be shared quickly (95%, 42/44). In Ghana, where health workers had less SMS experience, 55% (16/29) reported that it was easy to report by SMS with 72% identifying the speed of information sharing as its most beneficial feature (21/29). Locating patients, particularly hydrocele patients, was reported by both groups to be more difficult than the SMS reporting. However, during a focus group discussion in Ghana, they reported that the exercise enabled them to form stronger relationships with the LF patient community. The quotes below were provided by health workers during these two field tests as part of a semistructured questionnaire:

The community has been very happy with the programme and enjoyed it. There is great linkage between HSA (health surveillance assistant) and the community.

Malawi

It is fast information and it is easy to get good data in our catchment areas.

Malawi

The programme is very important because these patients will be assisted accordingly, it is encouraging relationship between HSA and the community.

Malawi

(The) community will profit from this method of surveillance.

Ghana

Now we know the cases, something can be done to help them.

Ghana

The MeasureSMS-Morbidity tool has recently been implemented in Dar es Salaam, Tanzania (approximately 4.5 million people), in order to test it in an urban environment and to further demonstrate its scalability. To facilitate this, a cascade training approach was adopted in which local CDDs identified cases using a house-to-house approach, and then CDD supervisors collated this information daily and reported it via SMS. This was the first survey in which the LF program staff used the MeasureSMS-Morbidity webpages to fully supervise the data being reported in real-time. Program staff have expressed their satisfaction in the tool as it was easy to set up and train health workers and it allowed them to instantly access the reported data via the Web, monitor the quality of the data, and provide feedback on the results to interested parties in a very short time frame. Preliminary data from this urban survey indicate a high LF morbidity burden in Dar es Salaam, with approximately 2000 lymphedema cases and 4000 hydrocele cases reported across the city. In recent months, additional rural implementations have been undertaken in parts of Ethiopia (covering approximately 450,000 people) and Malawi (covering approximately 0.5 million people), and a second urban application has been undertaken in Dhaka, Bangladesh (covering approximately 2.5 million people). Such a rapid increase in a short period of time clearly highlights that the tool is simple enough to be scalable across different settings.

Most importantly, these mapping tools help ministries of health establish the prevalence of disease in a given area, which is an essential component of the WHO dossier requirements [4]. These tools help LF programs prioritize, plan, and start treatment and care for patients. This is best exemplified in Malawi where hydrocele surgery camps and home-based lymphedema training started within months of the mapping being completed in the 2 most endemic districts of the country. In total, approximately 1850 hydrocele and 650 lymphedema cases were identified, and the Ministry of Health, with the support of LSTM through the Department of International Development UK funding, is now implementing an essential minimum care package. The distribution and use of this package was greatly enhanced through the mapping data, which informed where and what resources were required to implement the hydrocele surgeries and also to increase the training of health workers, community volunteers, and patients in home-based lymphedema care. Over the next 6 months, it is expected that all those with LF-induced hydrocele in these 2 districts of Malawi will have had or been offered surgery, and all lymphedema patients will be able to receive care. Plans to address the specific needs of the more severe cases and how to scale up the new LyMSS tool (see below) in collaboration with other international partners are underway. This is an extraordinarily powerful example of empowering local communities.

Implementation challenges varied between settings. As demonstrated in Ghana, mobile phone ownership does not necessarily equate to a familiarity with using the phone’s SMS functionality; therefore, basic mobile phone training may be necessary in some areas. Poor mobile phone coverage has thus far proven to be a secondary issue in comparison to the lack of regular access to phone charging facilities, particularly in rural areas. This to date has delayed submission of data but not prevented it entirely; however, knowledge of the local telecommunication networks is vital to the success of this tool. For example, to date, the cost of sending an SMS message has been covered by LSTM. While the cost of a single message is nominal (eg, approximately £0.01 in Malawi), at scale it consumes a large proportion of the overall cost to complete the survey. To address this, the use of bulk prepaid SMS options, referred to as bundles, should be encouraged where possible. For example, in Malawi you can purchase 600 SMS messages for MK600 (approximately £0.50), resulting in very large savings. Prior to starting the survey, research should also be undertaken to select which mobile network the relay phone should use (if multiple networks are available), as network quality can vary substantially in the target area. To account for this, the MeasureSMS-Morbidity tool allows multiple relay phones to be used (and thus multiple networks), such that all information is collated in a single database regardless of which relay phone it was received by.

**Figure 6:**
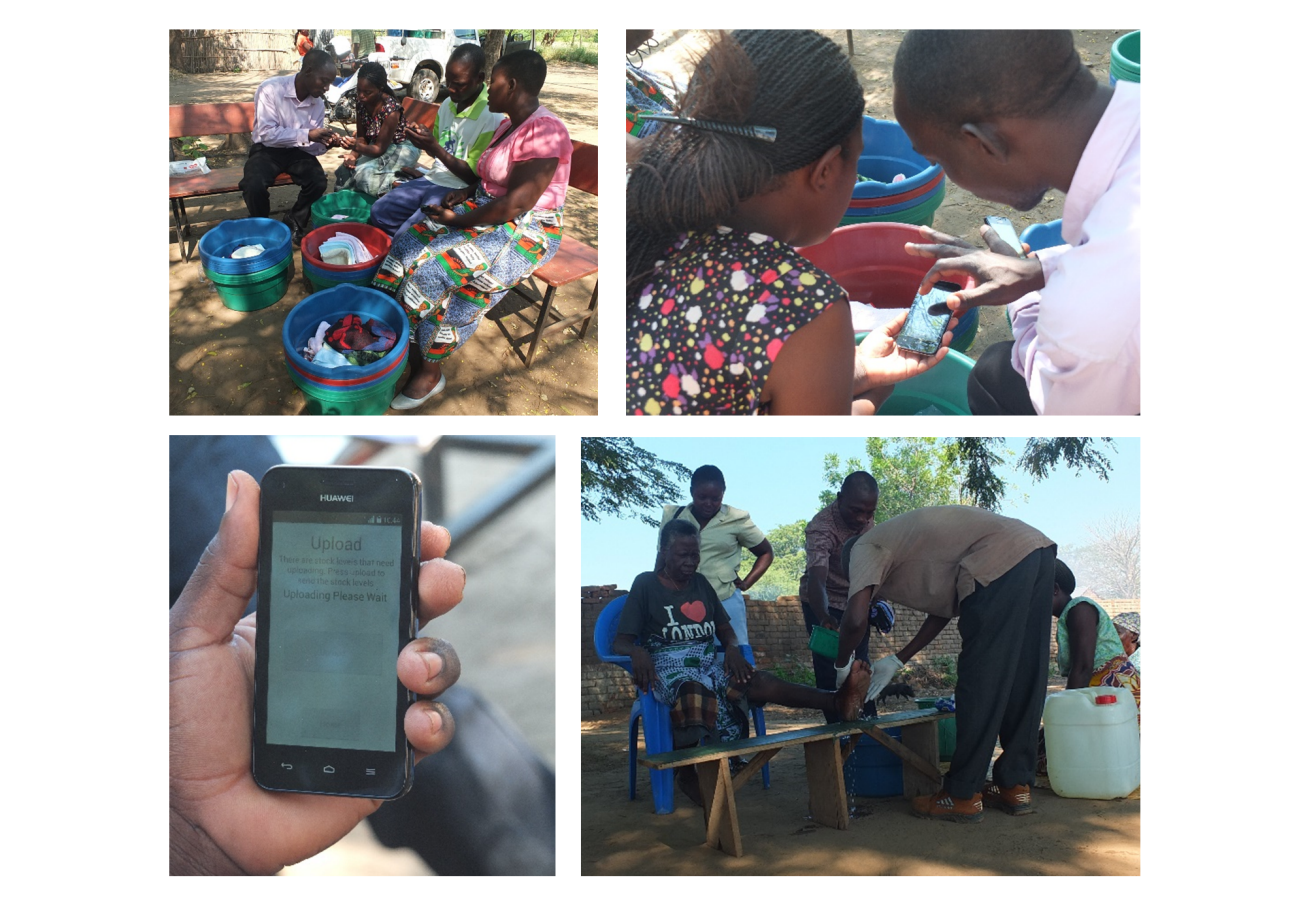
An example of the LyMSS tool in action.

The LyMSS tool is currently being tested in Chikwawa, Malawi (Figure 6). The LyMSS was set up in this area following the implementation of the MeasureSMS-Morbidity tool, which highlighted the prevalence of disease in the area. A total of 11 HSAs are involved, and since receiving initial training in May 2015 during which they were provided with a low-cost smartphone (eg, Huawei Ascend Y330, approximately £40), they have been continuously supplying 62 lymphedema patients with basic supplies, with soaps and antibacterial creams in highest demand. During the initial set-up, some problems were experienced in the local mobile network, with the data connection weak or non-existent in key areas (ie, around the health facility where the health workers gathered to send their initial supply reports). An interim follow-up in October 2015, however, indicated that this issue had been resolved, either through improvements in the local network or an increase in the health workers’ knowledge of areas where there was a sufficient signal. This was confirmed via a questionnaire, where only 2 of the 11 HSAs indicated that poor network had been an issue for them. A more pressing issue identified by the health workers was the issue of ensuring their phones were sufficiently charged in order to be able to send the reports. As much as 5 of the 11 HSAs did not have electricity in their homes, hence they generally used charging services at their local market. Phone credit was also identified as an issue as 8 of the 11 health workers had never used a smartphone prior to the study and were unfamiliar with mobile data tariffs. As with SMS, mobile data bundles were available for the chosen network (eg, 500MB for 30 days at MK2550, approximately £3), and during the training the HSAs were directed to use those. However, many HSAs chose not to consistently make use of the bundles over the 5-month period, resulting in higher costs and an increased likelihood of running out of phone credit.

Despite these perceived problems, the HSAs were not prevented from submitting their weekly reports, although they were occasionally submitted a day or 2 late. To date, over 500 soaps and 250 antibacterial creams have been distributed to patients over a 6-month period. The HSAs have expressed their personal satisfaction in being able to follow through and assist the patients, believing that regular contact with the patients has strengthened their relationship and encouraged the patients to open up about their problems and needs. During a focus group discussion, they also reported that sending supply reports by the smartphone app saved them time, although they did still need to communicate via telephone to arrange to collect additional supplies when needed. Improvements to the app were suggested by the HSAs, indicating that the scope could be extended for use beyond a simple supply monitoring tool.

The first version of the MeasureSMS-MDA tool has been developed and is being tested in December 2015 in Dar es Salaam, whereas the HealthFront health information tools are currently under development with plans to test the app in 2016.

## Discussion

### Principal Considerations

We advocate that community involvement and empowerment are essential for improving the health of many millions of people in underserved health-poor and resource-poor regions of the world. Community empowerment is the foundation of the LMS we describe here, and these tools have the potential to be applied to many other neglected tropical diseases and health conditions. The essential components of these tools include the simplicity of the data format, combined with the ease of its transference through a technology that is now a standard component of everyday life. This community-based approach sets it apart from more complicated, often research-oriented systems that have been applied elsewhere.

Further, these tools can be readily expanded to cover very large populations in a relatively short time frame, as we have demonstrated in our recent work in Africa with the MeasureSMS-Morbidity tool. The ability to scale up a tool and integrate it into existing national and subnational systems, structures, and policies is an essential part of mHealth initiatives, yet many fail to achieve this [17-19].

### Conclusion

As public health systems move more towards establishing universal health care for the populations of the developing countries [16], there will be a greater need for information obtained directly from local communities, many of which are geographically isolated. The role of new communication approaches driven by advances in mobile technology, such as the LMS described here, are therefore vital to improving the health of currently underserved populations.

## Abbreviations

CDD: community drug distributor
CHW: community health worker
HSA: health surveillance assistant
LMS: Liverpool mHealth suite
LSTM: Liverpool School of Tropical Medicine
LF: lymphatic filariasis
LyMSS: lymphedema management supply system
MDA: mass drug administration
SMS: short message service
WHO: World Health Organization
